# SLMFNet: Enhancing land cover classification of remote sensing images through selective attentions and multi-level feature fusion

**DOI:** 10.1371/journal.pone.0301134

**Published:** 2024-05-14

**Authors:** Xin Li, Hejing Zhao, Dan Wu, Qixing Liu, Rui Tang, Linyang Li, Zhennan Xu, Xin Lyu

**Affiliations:** 1 College of Computer and Information, Hohai University, Nanjing, Jiangsu, China; 2 Water History Department, China Institute of Water Resources and Hydropower Research, Beijing, China; 3 Research Center on Flood and Drought Disaster Reduction of Ministry of Water Resource, China Institute of Water Resources and Hydropower Research, Beijing, China; 4 Information Engineering Center, Yellow River Institute of Hydraulic Research, Yellow River Conservancy Commission of the Ministry of Water Resources, Zhengzhou, Henan, China; 5 Key Laboratory of Yellow River Sediment Research, MWR (Ministry of Water Resources), Zhengzhou, Henan, China; 6 Henan Engineering Research Center of Smart Water Conservancy, Yellow River Institute of Hydraulic Research, Zhengzhou, Henan, China; 7 Department of Orthopedics, The First Affiliated Hospital of Zhengzhou University, Zhengzhou, Henan, China; 8 School of Geodesy and Geomatics, Wuhan University, Wuhan, Hubei, China; Shandong Agricultural University, CHINA

## Abstract

Land cover classification (LCC) is of paramount importance for assessing environmental changes in remote sensing images (RSIs) as it involves assigning categorical labels to ground objects. The growing availability of multi-source RSIs presents an opportunity for intelligent LCC through semantic segmentation, offering a comprehensive understanding of ground objects. Nonetheless, the heterogeneous appearances of terrains and objects contribute to significant intra-class variance and inter-class similarity at various scales, adding complexity to this task. In response, we introduce SLMFNet, an innovative encoder-decoder segmentation network that adeptly addresses this challenge. To mitigate the sparse and imbalanced distribution of RSIs, we incorporate selective attention modules (SAMs) aimed at enhancing the distinguishability of learned representations by integrating contextual affinities within spatial and channel domains through a compact number of matrix operations. Precisely, the selective position attention module (SPAM) employs spatial pyramid pooling (SPP) to resample feature anchors and compute contextual affinities. In tandem, the selective channel attention module (SCAM) concentrates on capturing channel-wise affinity. Initially, feature maps are aggregated into fewer channels, followed by the generation of pairwise channel attention maps between the aggregated channels and all channels. To harness fine-grained details across multiple scales, we introduce a multi-level feature fusion decoder with data-dependent upsampling (MLFD) to meticulously recover and merge feature maps at diverse scales using a trainable projection matrix. Empirical results on the ISPRS Potsdam and DeepGlobe datasets underscore the superior performance of SLMFNet compared to various state-of-the-art methods. Ablation studies affirm the efficacy and precision of SAMs in the proposed model.

## 1 Introduction

Land cover classification (LCC) in remote sensing images (RSIs) is a fundamental task that quantitatively identifies various object types, including grassland, roads, buildings, water, and forests [1, 2]. The advent of deep learning and semantic segmentation has enabled pixel-level label generation for RSIs in an end-to-end automatic manner, meeting the demand for accurately interpreted images in practical applications like water resource management [3–5], precision agriculture [6–8], and hazard assessment [9, 10]. However, accurate surface environment evaluation through segmentation methods remains a challenge due to easily confused objects and discrete elements.

Semantic segmentation inherently involves three sub-problems: object recognition, localization, and boundary delineation [11–13]. Effectively addressing all these sub-tasks is essential for creating a robust network. Traditional approaches often rely on expert-designed feature extractors that struggle to adapt to complex and diverse scenarios [14]. In contrast, data-driven technologies such as deep convolutional neural networks (DCNNs) have made significant advancements [15–18]. Various DCNN-based models have gained prominence in remote sensing image (RSI) classification tasks [19, 20], with semantic segmentation playing a vital role in achieving detailed understanding.

The fully convolutional network (FCN) marked a breakthrough in computer vision by replacing fully connected layers with convolution layers, allowing an end-to-end trainable neural network for dense pixel predictions [21]. To address transformation loss during training and inference in FCNs, the encoder-decoder architecture, known as SegNet [22], was introduced to gradually recover the spatial dimensions of feature maps. Furthermore, U-Net, initially successful in medical images, was extended to remote sensing images (RSIs). FCNs, SegNet, and U-Net [23] have provided a solid foundation for subsequent research. However, their widespread adoption has been hindered by an inherent limitation—their structural rigidity, which restricts the utilization of informative and discriminative contexts.

We conduct a retrospective analysis of subsequently designed networks and classify them into two categories. The first category involves expanding the receptive field through dilated convolutions. For example, the DeepLab series [24–26] emerged by adjusting dilation rates to encompass more neighboring pixels in the convolution units. However, this approach leads to blurred edges where a large number of error-prone pixels are grouped together, negatively impacting the overall segmentation accuracy.

In contrast, attention mechanisms (AMs) have been developed as an alternative solution to address the diverse and complex variety of scenes. AMs aggregate more informative context, making various contextual dependencies quantifiable and obtainable. By injecting attentive features, pixels in both salient and unremarkable regions are better predicted. For instance, SENet [27] was designed to recalibrate channel-wise feature weights by modeling channel interdependencies, while NLNet [28] classifies video objects by capturing self-attentive correlations. Additionally, DANet [29] was explored by designing and integrating position and channel attention modules. However, these methods have limitations in terms of memory usage and processing time, making them significant concerns.

1. RSIs encompass a diverse array of complex ground objects, resulting in a sparse and imbalanced distribution. This high intra-class variation and inter-class similarity pose challenges for conventional networks. While attention mechanisms enhance the network’s capabilities by capturing and injecting pairwise correlations of pixels and channels, they also introduce memory usage and processing time overhead. Additionally, treating all paired pixels and channels equally may introduce noise with unnecessary dependencies, hindering feature optimization. Therefore, there is a need to selectively leverage the pairwise correlations of pixels and channels to enhance representations effectively.
2. Furthermore, feature aggregation during decoding is crucial for preserving fine-grained details and structural information. Conventional decoders often use bilinear upsampling to recover spatial dimensions, resulting in transformation loss. Moreover, insufficient fusion of multi-level representations suppresses favorable feature maps for dense prediction. Hence, the lossless aggregation of multi-level features in the decoder warrants further exploration.

To address the mentioned challenges, we propose two novel and efficient attention modules that selectively learn contextual affinities, resulting in attention maps with reduced computational overhead. The first module, named the Selective Position Attention Module (SPAM), leverages spatial pyramid pooling (SPP) [30] to resample feature anchors. The second module, the Selective Channel Attention Module (SCAM), aggregates channel-wise features and calculates affinities between each channel and the aggregated ones. Additionally, we introduce a multi-level feature fusion decoder that employs data-dependent upsampling (DUpsampling) [31] to recover and fuse features without loss. Our contributions can be summarized as follows:

1. We propose SPAM, which selectively learns spatial context. Specifically, SPAM calculates contextual affinities using SPP to extract multi-scale feature anchors, reducing complexity while enhancing distinguishability, particularly for sparse and imbalanced RSIs.
2. We propose a SCAM to selectively learning channel-wise contexts. More concretely, SCAM aggregates channel-wise contextual information by reducing feature maps to fewer channels, capturing channel-attentive dependencies without information loss.
3. We present the Multi-Level Feature Fusion Decoder (MLFD) for the aggregation of multi-level feature maps. MLFD utilizes DUpsampling to increase spatial dimensions with a learnable projection matrix, minimizing transformation loss while preserving spatial details.
4. We construct SLMFNet, incorporating the above modules, and evaluate its performance on two datasets: ISPRS Potsdam and DeepGlobe [32], which involve aerial and satellite images. We conduct both quantitative and qualitative comparisons and validate the efficiency and effectiveness of MLFD through an ablation study.

The remainder of this paper is organized as follows: Section 2 introduces related works. Section 3 presents the proposed framework and the pipeline of sub-modules in detail. Section 4 offers a quantitative and qualitative evaluation of SLMFNet. Finally, our conclusions are presented in Section 5.

## 2 Related works

### 2.1 Land cover classification by semantic segmentation of RSIs

Motivated by significant advances and the proliferation of updated CNN-based architectures [33–35], numerous transferable models have been applied to remote sensing images (RSIs) with heuristic enhancements. Each pixel in an RSI carries semantic information, encompassing various elements of the landscape and prominent objects such as rivers, farmland, vehicles, buildings, and more. Additionally, RSIs are captured from high altitudes, presenting two major challenges that a robust RSI segmentation network must confront. Several attempts have been made to enhance segmentation accuracy in RSIs.

Commonly employed post-processing techniques, such as boundary smoothing [36], have been utilized. These methods consider implicit spatial correlations through the gradual merging of neighboring segments, leading to more precise border labeling. Other approaches utilize pre-generated superpixels [37] or connected conditional random fields (CRFs) [38] to refine segments. Color-infrared images are intrinsically used as pairwise potentials for distance evaluation between pixels.

In addition to post-processing, end-to-end trainable networks hold practical significance. Numerous methodologies have been proposed to enrich contextual information in learned representations. For example, HRNet [39], introduced by [40], replaces the original encoder to generate high-resolution feature maps, demonstrating superiority on ISPRS benchmarks. Similarly, [41] proposed a self-cascaded network using dilated convolutions to learn multi-scale representations in the final layer of the encoder. ResUNet-a [42] combines multiple useful modules and strategies, integrating hierarchically contextual dependencies with skip connections. However, the multi-scale feature extraction and fusion strategy in these approaches still offer room for improvement, and the computational complexity has been criticized.

Recent research has explored attention mechanisms (AMs) to creatively model long-range dependencies using matrix computations [43]. The computed attention map can be flexibly integrated into raw features, enhancing the distinguishability of easily confused objects.

Empirically, AMs function as selectors that enable the network to focus on essential words, regions, or relationships. The original application of AMs was in machine translation tasks [44], where they established global dependencies between input and output features. In the context of semantic segmentation tasks, AMs perform a similar role in emphasizing significant components. A fundamental development in this domain is SENet [27], which highlights channels of feature maps with higher weights after learning global pooling features. Extending the channel-wise SE operator to the spatial domain, CBAM [45] underscores meaningful sub-regions. On the other hand, the non-local neural network (NLNet) [28] concurrently learns multiple dimensions’ contexts for visual tasks. The self-attention concept also inspired OCNet [46], which extracts object-level contextual information to aid in object recognition. CCNet [47] recurrently stacks two crisscross AMs. Another notable attention-based network, DANet [29], was introduced in 2019. This approach enables AMs to adaptively couple position-wise dependencies and emphasizes intra-dependencies between channels. The parallel outputs are then summed up for the decoder stage. Although DANet remarkably captures and incorporates informative context, it introduces significant computational complexity.

Recently, many semantic segmentation networks for RSIs with AMs have been developed [48, 49]. For instance, Teerapong et al. [50] employed a channel attention block to enhance RSI segmentation accuracy and constructed a transferable learning model. Additionally, three representative methods are elaborated. The first one is SCAttNet [51], designed to learn an attention map to adaptively aggregate contextual information for each point in RSIs. Alongside local context analysis, LANet [52] bridges the gap between high-level and low-level features, and the designed patch AMs optimize the representations compatibly. Similarly, [53] introduced a hybrid multiple attention network for aerial image semantic segmentation, enabling the network to adaptively learn spatial, channel, and class correlations to enhance the distinguishability of learned representations. Concerning the imbalanced distributions of RSIs, Zhou et al. [12] presented a novel dynamic weighting method based on effective sample calculation for semantic segmentation in remote sensing, significantly improving minimal-class accuracy and recall in imbalanced datasets, as demonstrated in diverse applications like forest fire area segmentation and land-cover semantic segmentation using the Landsat8-OLI and LoveDA datasets. Likewise, Li et al. [54] proposed a novel SSCNet that integrates spectral and spatial information using a joint spectral–spatial attention module (JSSA), significantly enhancing semantic segmentation in RSIs, as demonstrated by superior performance on ISPRS Potsdam and LoveDA datasets and validated through comprehensive ablation studies.

In contrast to existing networks, our primary objective is to reduce computational complexity while maintaining segmentation accuracy. Building on the concept of using complementary context, DANet has proven to be a simple yet effective approach. Motivated by the resampling of spatial feature anchors and the aggregation of feature channels, we propose two lightweight AMs to learn contextual affinity and enhance the distinguishability of learned representations. This significantly reduces the number of matrix multiplications while effectively learning contextual information. In the remainder of this section, we introduce and analyze the DANet pipeline, including the position attention module (PAM) and channel attention module (CAM).

### 2.2 Revisiting dual attention modules

DANet [29] considers various scales of stuff and objects, occlusions and illuminations as direct interferences that limit the discriminability. Hence, PAM and CAM are designed to refine representations by enriching contextual information.

**Fig 1:**
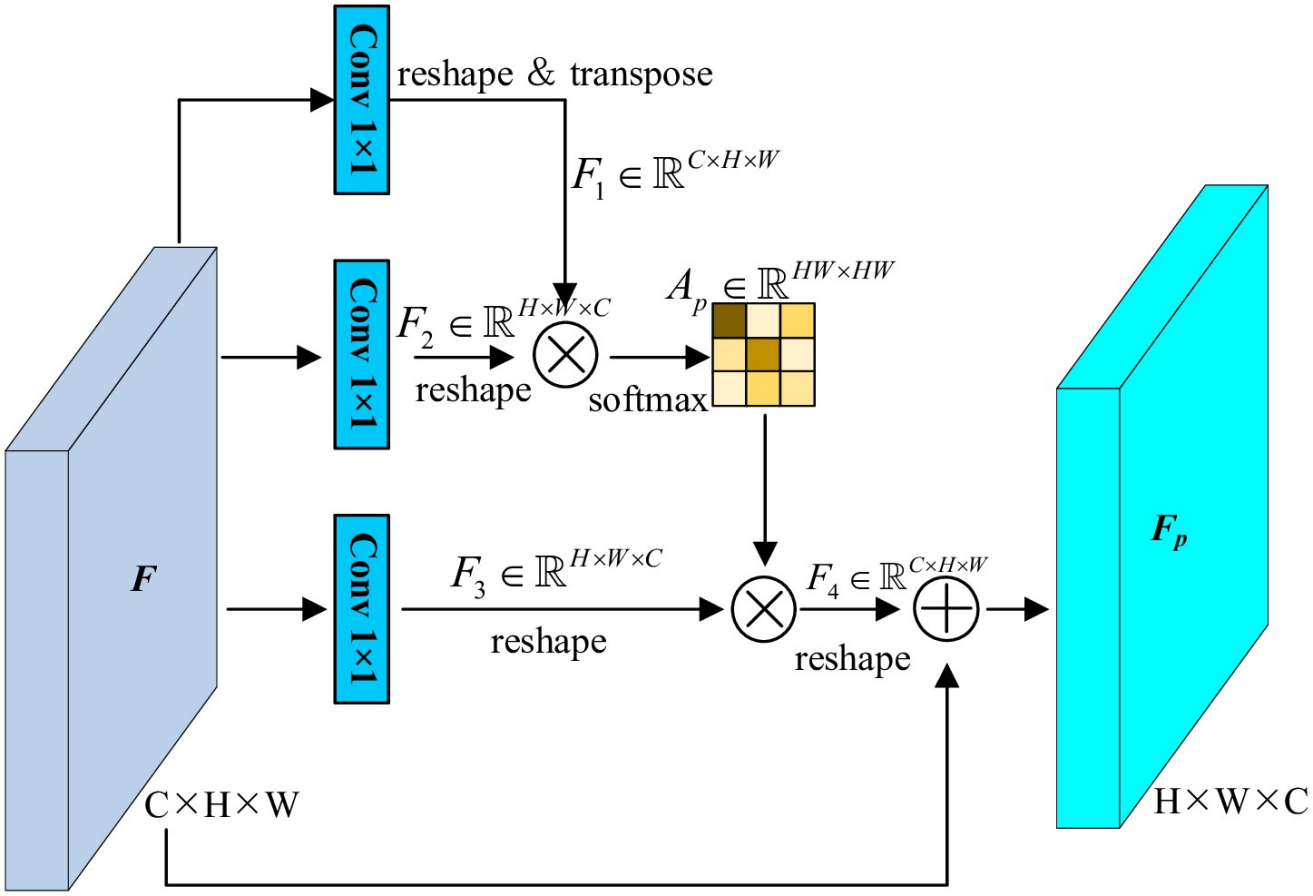
Pipeline of PAM.

Fig 1 presents pipeline of PAM. Given the input feature $F\in R^{C\times H\times W}$, three convolved features are generated and keep the par same size with the input. In this top branch, the feature is then reshaped and transposed, producing $F_{1}\in R^{N\times C}$, where *N* = *H* × *W* denotes the number of pixels. And $F_{2}\in R^{C\times N}$ is also flatterned. By the matrix multiplication followed by a softmax layer, the position attention map is calculated,

$$\begin{array}{c} A_{p}(i,j)=\frac{\;\text{exp}\;(F_{1}(i)\cdot F_{2}(j))}{\sum_{i=1}^{N}\;\text{exp}\;(F_{1}(i)\cdot F_{2}(j))} \end{array}$$ (1)

where *A*_*p*_(*i*, *j*) measures the inter-impacts between pixels. Essentially, the correlations contribute more to representations if the similarity is higher. Then the last two branches are used to produce position-wise attention injected features. Formally,

$$\begin{array}{c} F_{p}=\mu \sum_{i=1}^{N}(A_{p}(j,i)\cdot F_{3})+F_{5} \end{array}$$ (2)

where *μ* is a learnable coefficient which is initialized as 0.

**Fig 2:**
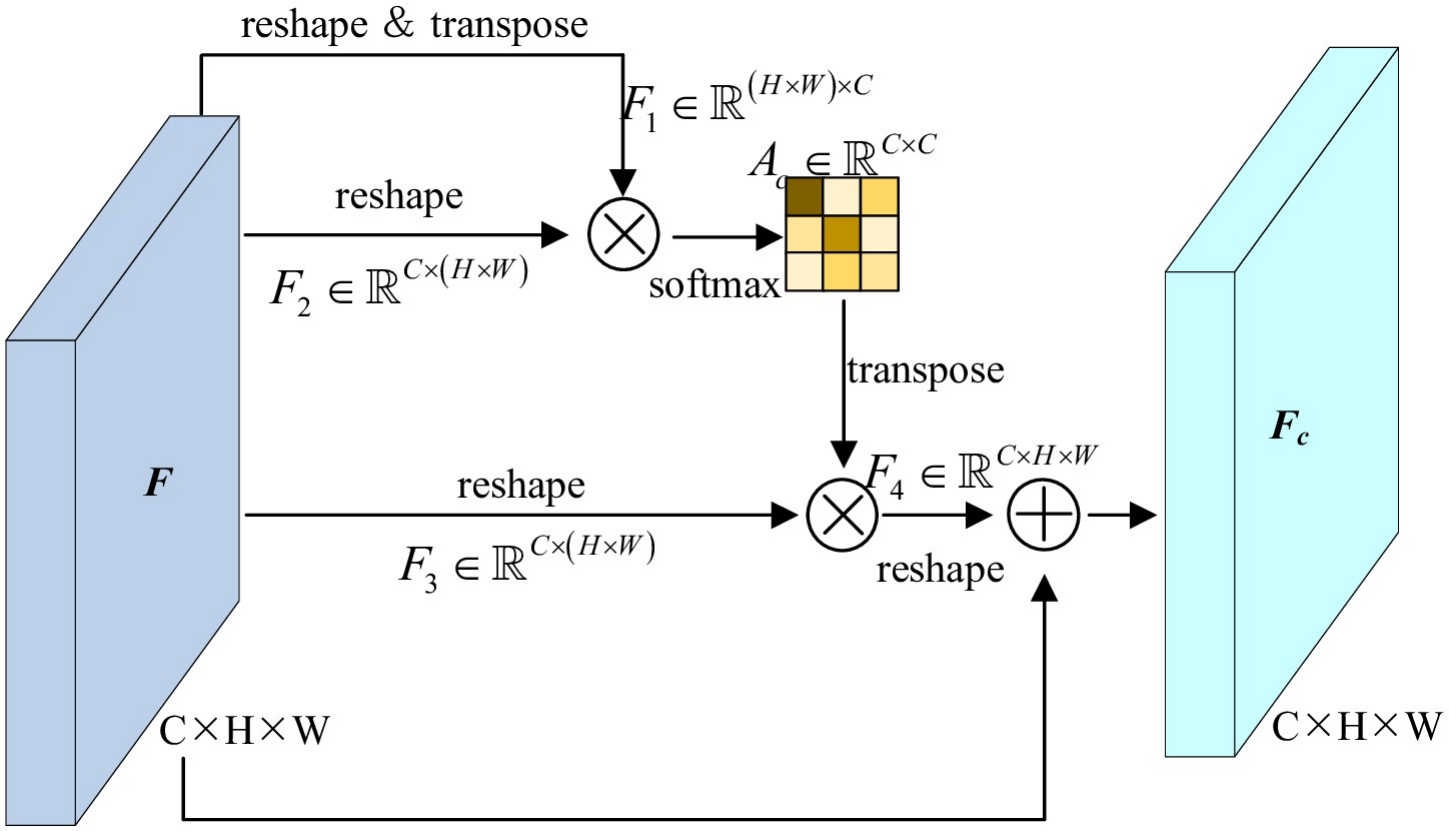
Pipeline of CAM.

CAM has a semblable multiple-branching structure. The input feature $F\in R^{C\times H\times W}$ is initially feed into the calculation of attention map as shown in Fig 2. The reshaped and transposed $F_{2}\in R^{C\times N}$ is left multiplied with $F_{1}\in R^{N\times C}$, where *N* = *H* × *W*. Therefore, the channel attention map is formed as,

$$\begin{array}{c} A_{c}(i,j)=\frac{\;\text{exp}\;(F_{1}(j)\cdot F_{2}(i))}{\sum_{i=1}^{C}\;\text{exp}\;(F_{1}(j)\cdot F_{2}(i))} \end{array}$$ (3)

where $A_{c}(i,j)\in R^{C\times C}$ accumulates the channel-wise inter-impacts. Then the output feature maps can be refined by,

$$\begin{array}{c} F_{c}=\gamma \sum_{i=1}^{C}(A_{c}\cdot F_{3})+F_{5} \end{array}$$ (4)

where *γ* is a scale parameter. The element-wise summation of channel attentive features and raw input features is defined as the enhanced ones with the injection of channel-wise relationships.

In summary, DANet preferably captures and leverages the spatial and channel inter-dependencies that consider all the positions and channels. Although the representations are refined significantly, a mass of computations supervenes for calculating attention map. Inherently, not all the positions or channels are indispensable for boosting the network’s discernibility. Moreover, the sparsity and imbalanced distribution of RSI are exceptionally salient, making DANet inefficient.

## 3 The proposed method

### 3.1 Framework

**Fig 3:**
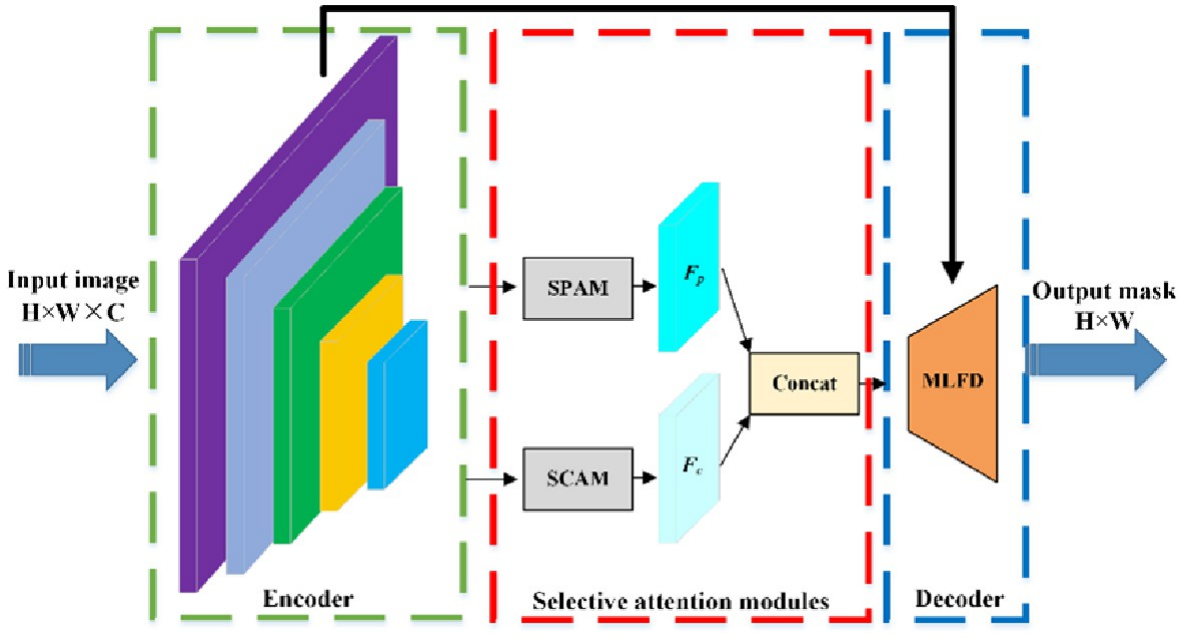
Overall framework.

As illustrated in Fig 3, SLMFNet strives to refine learnt representations for RSI semantic segmentation. Although attention mechanisms have revealed a strong capability in modeling contextual information in spatial and channel domains, the redundant computations are supervened due to the stereotype of equally handling coupled pixels and channels. Then, our investigation shows that resampling informative and representative feature anchors and channels enable the AMs to collect satisfactory contextual cues. Therefore, SPAM and SCAM are designed and embedded at the end of feature encoder. Subsequently, MLFD is formed to recover the spatial resolution losslessly, ensuring reconstructed representations undistorted.

### 3.2 Selective position attention module

**Fig 4:**
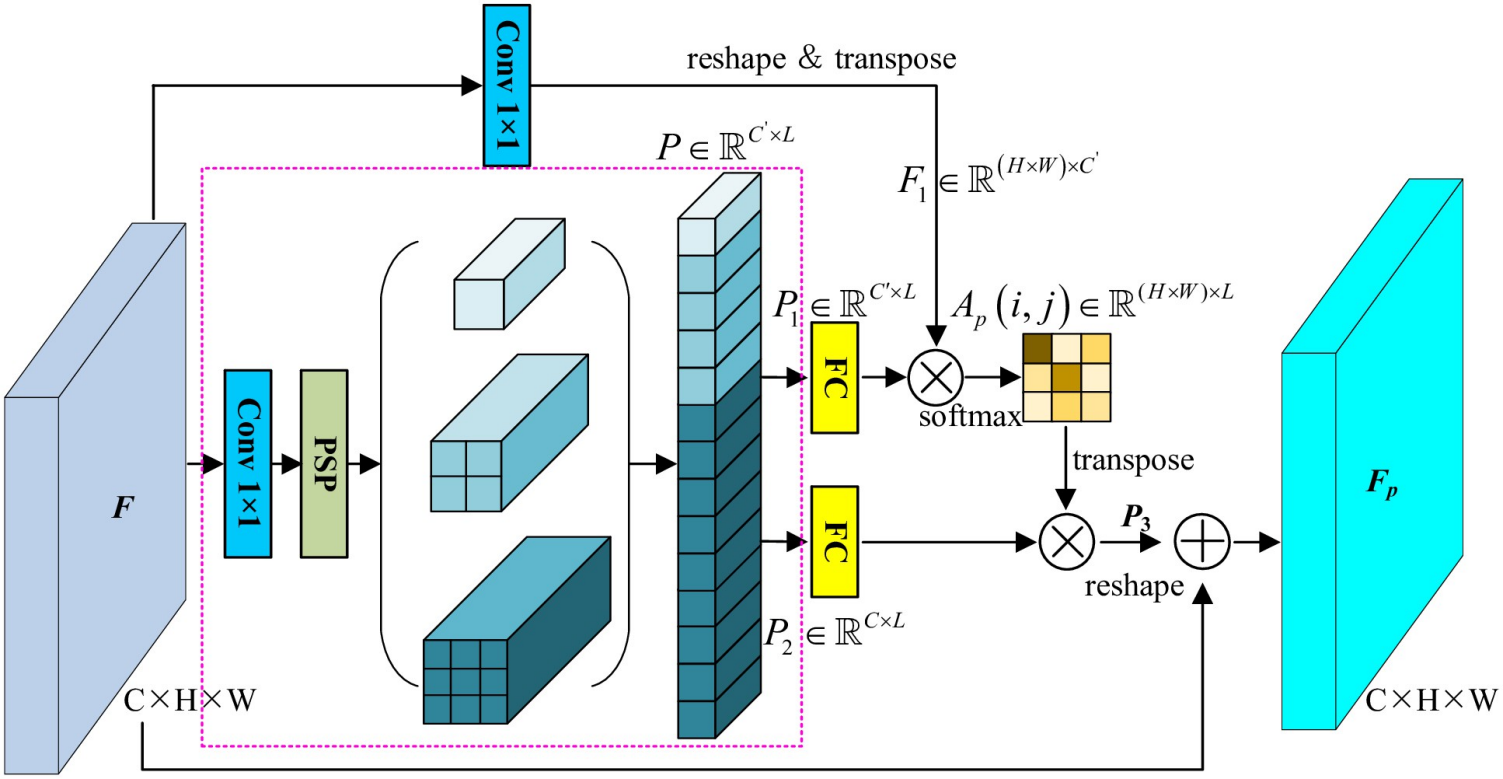
Pipeline of SPAM.

As discussed above, the original PAM models the dependencies of all pairwise pixels. Moreover, the correlations are implemented by the vectorial internal product, which occupies huge GPU memory and costs much more time with massive pixels. Thus, to our analysis, not all pairwise relationships are meaningful in contributing to the completeness and uniqueness of a specific pixel’s representation. However, concerning the sparsity and variety of the covered objects in RSI, resampling the representative positions as the feature anchors, which are then utilized to model position-wise spatial attention map, paves an artful way to inject sufficient spatial dependencies for feature optimization. Thus, the pipeline is designed in Fig 4.

Concretely, given the input feature map $F\in R^{C\times H\times W}$, where *C*, *H*, *W* indicate the number of channels, height and width. Similarly, three parallel branches are presented, in which the middle branch is the key. After a 1 × 1 convolution layer, SPP at three scales is applied. The elements in pooled features are viewed as feature anchors, also known as the gathering centers. Then the pooled features are concatenated and flattened to vectors $P\in R^{C^{\prime }\times L}$, where *C*′ denotes the channel value of rebuilt feature maps, and *L* is the sum of feature anchors in all size of pooled features. Therefore, two fully connected layers are used to build two independent representations of feature anchors with $P_{1}\in R^{C^{\prime }\times L}$ and $P_{2}\in R^{C\times L}$. Commonly, the position attention map is obtained by matrix multiplication and softmax layer,

$$\begin{array}{c} A_{p}(i,j)=\frac{\;\text{exp}\;(P_{1}(j)\cdot F_{1}(i))}{\sum_{i=1}^{L}\;\text{exp}\;(P_{1}(j)\cdot F_{1}(i))}\;, \end{array}$$ (5)

where $A_{p}(i,j)\in R^{(H\times W)\times L}$ measures the correlation between the *j*th pixel and *i*th anchors, and $F_{1}\in R^{\left(H\times W\right)\times C^{\prime }}$, marked as the top branch in Fig 4, is reshaped and transposed features after convolved with kernel size of 1 × 1. It is identical that *F*_1_ keeps the same channels to *P*_1_. Afterward, the attention map *A*_*p*_(*i*, *j*) is transposed to $A_{p}^{T}(i,j)\in R^{L\times (H\times W)}$. Thereby, *P*_3_ is formed as below,

$$\begin{array}{c} P_{3}=P_{2}\cdot A_{P}^{T} \end{array}$$ (6)

where $P_{2}\in R^{C\times L}$ denotes the representation of feature anchors, and $A_{p}^{T}(i,j)\in R^{L\times (H\times W)}$ derives from attention map. Finally, the reshaped $P_{3}\in R^{C\times H\times W}$ is element-wise summed with input feature maps *F* to output refined representations *F*_*p*_. And the calculation can be expressed as follow,

$$\begin{array}{c} F_{p}=\mu P_{3}+F \end{array}$$ (7)

where *μ* is a learnable coefficient (initially set to 0.5). $F_{p}\in R^{C\times H\times W}$ is the refined representations of SPAM.

To evaluate the complexity, we quantify the magnitude of computational complexity. As for PAM, the computational complexity is determined as

$$\begin{array}{c} o(C^{\prime }H^{2}W^{2})=o(C^{\prime }N^{2}) \end{array}$$ (8)

where *H*, *W* and *C*′ refer to the input feature’s spatial size, *N* = *H* × *W* represents the flattened tensor dimension. By contrast, SPAM only levelled down the complexity to *o*(*C*′*NL*), where *L* ≪ *N*. Taking four branches for spatial pyramid pooling with kernel size of 1 × 1, 2 × 2, 3 × 3, and 6 × 6, fifty spatial anchors are resampled. Given the input *H* × *W* = 256 × 256, the complexity ratio between PAM and SPAM is,

$$\begin{array}{c} r=\frac{o(C^{\prime }N^{2})}{o(C^{\prime }NL)}=\frac{256\times 256}{50}\approx 1311 \end{array}$$ (9)

Consequently, there are about 1311 times matrix multiplications are saved by SPAM.

Apart from the original PAM, SPAM first resamples feature anchors, gathering neighboring pixels by spatial pyramid pooling at various scales. Subsequently, the position attention map is calculated between every pixel and feature anchors instead of pairwise pixels. Also, the dependencies along with spatial domain are injected into raw representations.

### 3.3 Selective channel attention module

**Fig 5:**
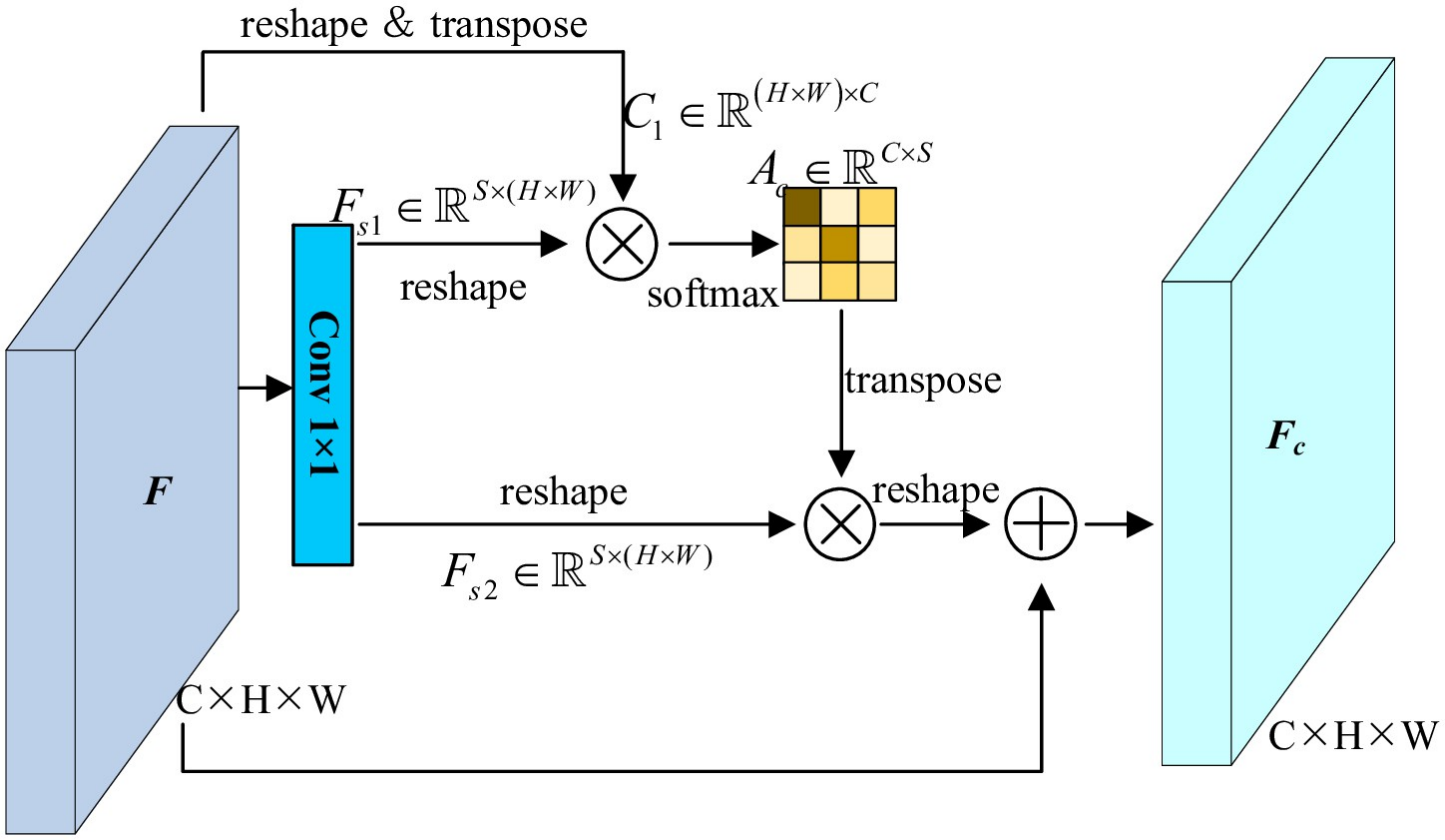
Pipeline of SCAM.

As for CAM, the dependencies stem from channel-wise correlations. Practically, the existing CAMs calculate the correlations between all channels, neglecting the informativeness of specific channel. Meanwhile, the computational burden is brought in. As illustrated in Fig 5, SCAM is devised with initially shrinking the number of feature channels. In our opinion, the aggregated channels are representative and informative are capable of providing satisfactory feature clues. Then, the channel-wise dependencies are collected between each channel and the shrunk ones.

Given an input feature termed as $F\in R^{C\times H\times W}$, where *C*, *H*, *W* indicate the number of channels, height and width. Considering the middle branch, a 1 × 1 convolution layer is applied to *F* at first. Then reshaping convolved features to generate $F_{s1}\in R^{S\times (H\times W)}$ and $F_{s2}\in R^{S\times (H\times W)}$, where *S* indicates the shrunk number of channels and *S* ≪ *C*. Therefore, the selective channel attention map is computed between $F_{s1}\in R^{S\times (H\times W)}$ and $C_{1}\in R^{(H\times W)\times C}$ (the reshaped and transposed raw feature). Formally,

$$\begin{array}{c} A_{c}(i,j)=\frac{\;\text{exp}\;(C_{1}(j)\cdot F_{s1}(i))}{\sum_{i=1}^{S}\;\text{exp}\;(C_{1}(j)\cdot F_{s1}(i))} \end{array}$$ (10)

where *F*_*s*1_(*i*) denotes the *i*’th representative channel of *F*_*s*1_, *C*_1_(*j*) denotes the *j*’th channel of raw feature *C*_1_. The *A*_*c*_(*i*, *j*) quantifies the channel-pair impacts and $A_{c}\in R^{C\times S}$ denotes the selective channel attention map. In essence, the boosted channel-wise representation has been injected the correlation with resampling representative and informative channels. After capturing channel attention map, the output is naturally formed as,

$$\begin{array}{c} F_{c}=\gamma \sum_{i=1}^{S}(A_{c}\cdot F_{s2})+F \end{array}$$ (11)

where *γ* is a learnable coefficient (initially set to 0), and $F_{c}\in R^{C\times H\times W}$ is the output of SCAM.

To evaluate the complexity, we quantify the magnitude of computational complexity by calculating the matrix multiplication times. Definitely, the shrunk channels *S* is far from original ones *C*. The ratio of complexity comparison is perceived as,

$$\begin{array}{c} r=\frac{o(C^{2})}{o(CS)} \end{array}$$ (12)

As analyzed above, reducing the number of channels results in a reduction in the number of matrix multiplications, thereby allowing the network to convey its intended information more efficiently.

Note that the convolution layers are not embedded initially due to maintaining the channel-wise relationships. Following the theoretical derivation, SCAM allows fewer computations to capture sufficient channel-wise dependencies. Then an immediate yet practicable skip connection is deployed to fuse the modeled channel correlations.

### 3.4 Multi-level feature fusion with data-dependent upsampling

On the question of recovering learnt features to original spatial size, a sophisticated fusion strategy is urgently needed for aggregating multi-level features, positively exerting the segmentation results. Two features sources, the encoded one from encoder phases and the decoded one from decoder phases, contribute to enhancing distinguishability and preserving geo-objects’ details in gradually expanding procedure. However, the commonly used bilinear upsampling inevitably causes information loss. Hence, we propose a MLFD, replacing bilinear upsampling with DUpsampling. Besides, the encoded features are fused with decoded ones via skip connections.

**Fig 6:**
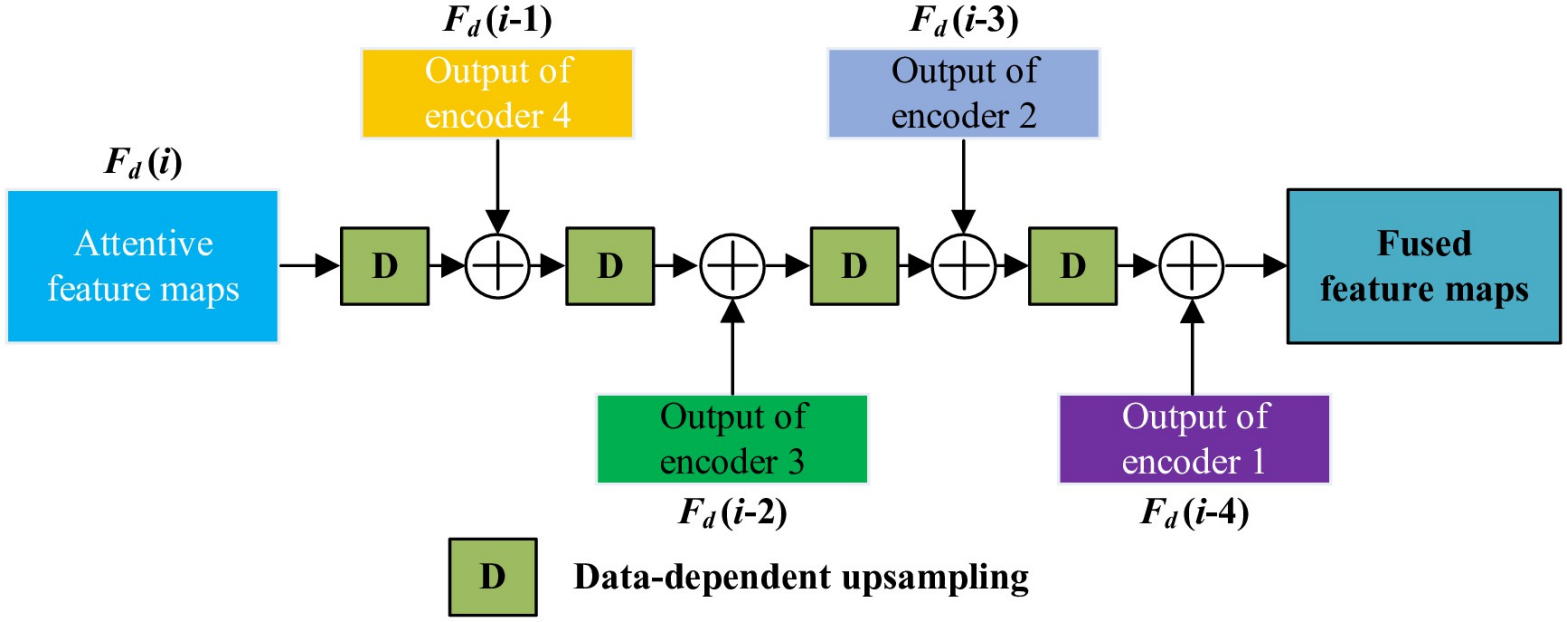
Architecture of MLFD.

As shown in Fig 6, the flows are presented. The input attentive feature maps derive from the output of SAMs which marked in the red dotted box in Fig 3. Here, this input is denoted as *F*_*d*_(*i*), where *i* represents the order of encoder block. In our network, *i* is positive integer and pre-defined as 5. And we re-define the original spatial size of image is *H* × *W* (channels are irrespective). Then, the ratio of different scales of features to original spatial size can be formed as,

$$\begin{array}{c} F_{d}^{s}(i)=\frac{H}{2^{i-1}}\times \frac{W}{2^{i-1}} \end{array}$$ (13)

where $F_{d}^{s}(i)$ represents the spatial size of *F*_*d*_(*i*), and *H* × *W* is the spatial size of raw image. Therefore, a fusion process of neighboring features can be represented as,

$$\begin{array}{c} F_{d}^{f}(F_{d}(i),F_{d}(i-1))=F_{d}(i-1)⊕f_{d}(F_{d}(i)) \end{array}$$ (14)

where $F_{d}^{f}$ is the fused feature of two neighboring phases, ⊕ denotes the element-wise summation, and *f*_*d*_(⋅) represents the data-dependent upsampling. For the sake of understanding, the necessary explanation of DU is presented. [31] revealed that the ground truth mask preserves enough mutual dependent structural information and can be compressed with arbitrary ratio losslessly. As a consequence, they developed DU based on the rationale of matrix projection. Explicitly, a learnable transformation matrix is devised as the projection coefficient for changing spatial size. And the matrix is unceasingly tuning by minimizing the following loss function,

$$\begin{array}{c} L_{D}(i)=\underset{M_{c}}{\text{argmin}}{∥g_{i-1}-M_{c}(F_{d}(i))∥}^{2} \end{array}$$ (15)

where *M*_*c*_ is the transformation matrix, *g*_*i*−1_ represents the compressed ground truth mask with the same spatial size of *F*_*d*_(*i* − 1), and *M*_*c*_(*F*_*d*_(*i*)) means data-dependent upsampling of *F*_*d*_(*i*).

Finally, the gradually recovered feature maps, also known as fused feature maps in Fig 6, reach the spatial size of *H* × *W*. Specifically, the DUpsampling procedure is seamlessly embedded into the decoder as a 1 × 1 convolution, without heavy computations.

## 4 Experiments and discussions

### 4.1 Datasets

To evaluate the proposed method, we conduct the experiments on two benchmarks that derive from different platforms with different properties.

#### 4.1.1 ISPRS Potsdam benchmark dataset

**Fig 7:**
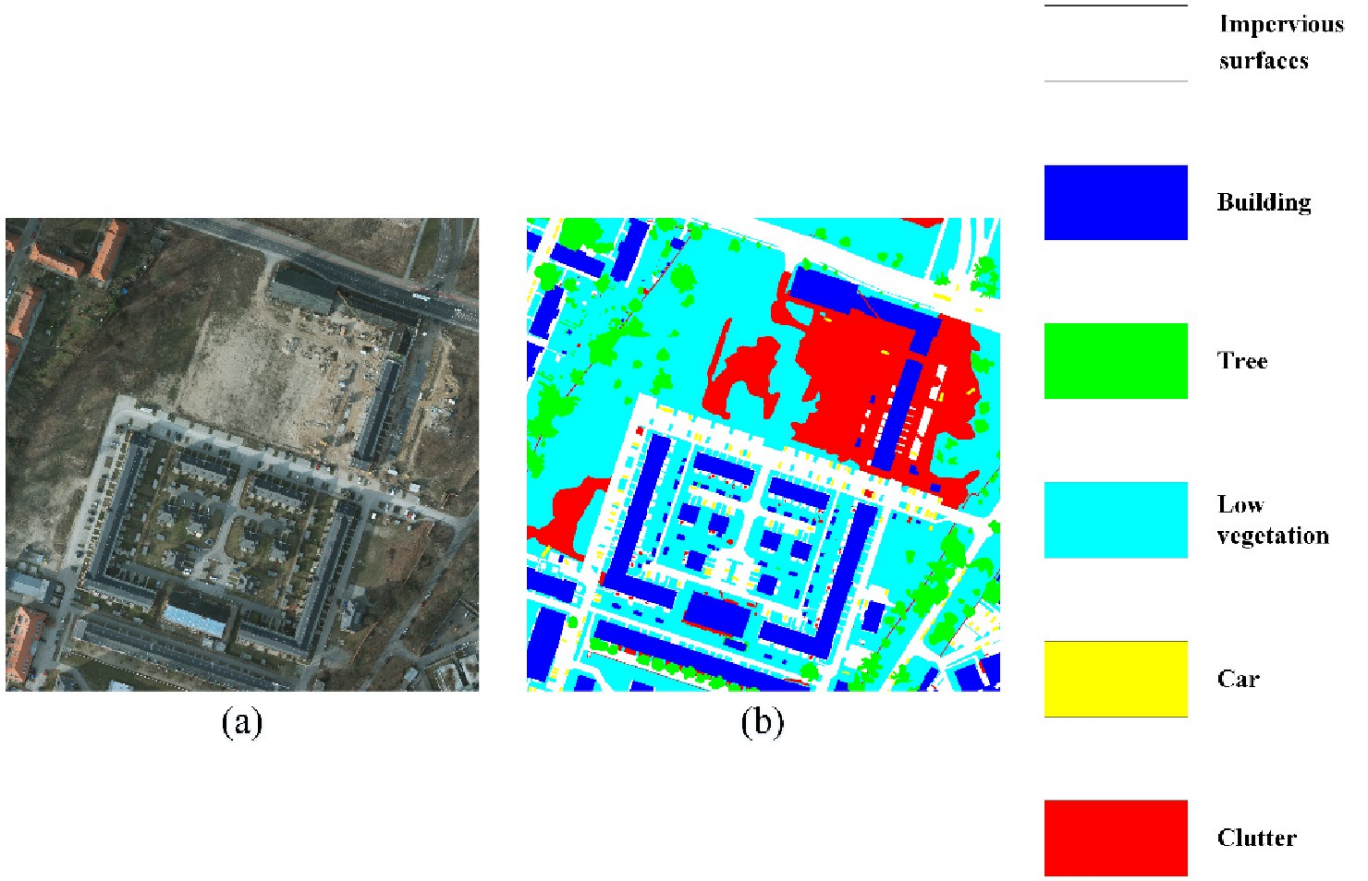
Illustration of Potsdam dataset.

The ISPRS Potsdam benchmark dataset is acquired from aerial platform, with a coverage of Potsdam, Germany. The associated ground truth masks are labeled with six land cover categories: impervious surfaces, buildings, low vegetation, trees, cars and clutter. There are 24 images available with a spatial size of 6000 × 6000 pixels. The spatial resolution is about 5 cm. One sample is illustrated in Fig 7.

#### 4.1.2 DeepGlobe land cover classification dataset

**Fig 8:**
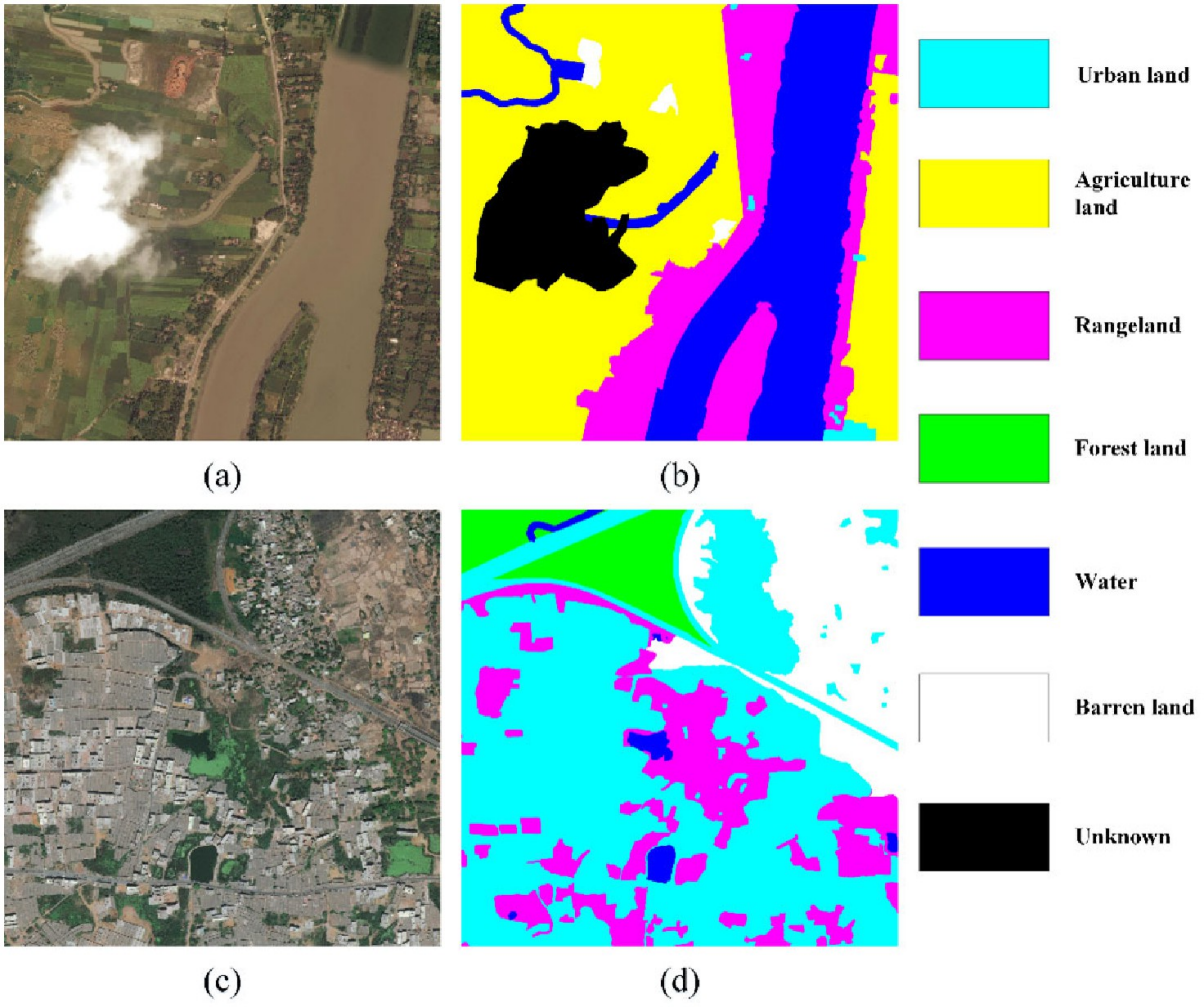
Illustration of DeepGlobe dataset.

The DeepGlobe Land Cover Classification Dataset offers sub-meter satellite imagery concerning three cities, Vegas, Potsdam, and Paris. Apart from aerial imagery, the satellite imagery suffers more from diversity and variety of land cover stuff. There are 1146 images with a spatial size of 2448 × 2448 pixels and spatial resolution of 0.5 m. The paired masks are annotated with seven categories: urban land, agriculture land, rangeland, forest land, water, barren land and unknown. One sample is illustrated in Fig 8.

### 4.2 Implement details

The proposed SLMFNet is implemented under PyTorch framework. All the models were implemented using a single Nvidia A40 GPU with 40GB memory under Linux OS for experiments. Table 1 lists the hyper-parameters. The backbone of the encoder is ResNet 101, which is marked in the green dotted box in Fig 3. In this study, we did not employ pretraining on additional data sources for the ResNet 101. For both Potsdam and DeepGlobe datasets, the settings are the same.

**Table 1:** Hyper-parameter settings.

Items	Settings
Backbone	ResNet 101
Learning strategy	Poly decay
Initial learning rate	0.002
Loss function	Cross-entropy
Optimizer	Adam
Max epoch	500
GPU memory	40 GB
Sub-patch size	256 × 256
Batch size	32

The available data is split into sub-patch with a spatial size of 256 × 256. The data properties and partitions are then presented in Table 2. The data partitions satisfy a ratio of 8:1:1. And the same data augmentation strategies are carried.

**Table 2:** Data properties and partitions.

Datasets	Potsdam	DeepGlobe
Bands	NIR, R, G	R, G, B
GSD	5 cm	0.5 m
Total number of sub-patches	8576	65043
Number of training	6860	52035
Number of validation	858	6504
Number of test	858	6504
Data augmentations	Rotate 90, 180, and 270 degrees, horizontally and vertically flip	

At last, the comparative methods are listed in Table 3. FCN, SegNet, U-Net and DeepLab V3+ are the pioneering segmentation networks. Then the attention-based networks are involved, including NLNet and OCNet. For RSI segmentation, ResUNet-a, SCAttNet, HMANet and LANet are reproduced. Besides, DANet, the prototype of our SLMFNet, is compared.

**Table 3:** Comparative methods.

Methods	Abstracts
FCN-8s [21]	Fundamental segmentation networks in CV
SegNet [22]	
U-Net [23]	
DeepLab V3+ [26]	
NLNet [28]	Representative attention-based segmentation networks in CV
OCNet [46]	
ResUNet-a [42]	A hybrid segmentation network for RSI
SCAttNet [51]	Attention-based networks for RSI
HMANet [53]	
LANet [52]	
DANet [29]	Prototype of SLMFNet
SLMFNet	Ours

### 4.3 Numerical metrics

Pixel-wise intersection over union (mIoU) and overall accuracy (OA) are opted as the evaluation metrics. The formulas are as follows,

$$\begin{array}{c} OA=\frac{TP+TN}{TP+FP+FN+TN} \end{array}$$ (16)

$$\begin{array}{c} mIoU=\frac{TP}{TP+FP+FN} \end{array}$$ (17)

where TP, FP, FN and TN represent the number of true positives, the number of true positives, the number of false negatives, and the number of true negatives, respectively.

### 4.4 Compared with State-of-the-art methods

#### 4.4.1 Results of Potsdam dataset

Since contextual information and multi-level feature fusion are important, SLMFNet is proposed to flexibly capture spatial and channel context and losslessly fuse multi-level features. This section collects the accuracy of the ISPRS Potsdam test set, experimentally evaluating SLMFNet.

Table 4 reports the results of the Potsdam test set, including class-wise and overall performance. First, the class-wise IoU and pixel accuracy are counted. Then the mIoU and OA are calculated. In general, the results evident that SLMFNet outperforms comparative models by a remarkable margin. As discussed previously, SAMs boost efficiency while retaining robust in providing context-enhanced features. Moreover, the MLFD decoder is devised to improve the learnt representations for inference during decoding. Therefore, a considerable amount of mIoU and OA is obtained by SLMFNet, scoring more than 65% and 89%.

**Table 4:** The results of the Potsdam test set with class-wise performance in form of IoU/OA and overall performance with mIoU and OA, where bold indicates the best.

Methods	Impervious surfaces	Building	Low vegetation	Tree	Car	Clutter	mIoU	OA
FCN-8s [21]	67.37/80.01	64.67/85.17	53.05/72.77	49.52/68.05	43.66/66.67	29.11/43.18	51.23	69.31
SegNet [22]	69.46/83.71	66.68/89.11	54.70/76.13	51.06/71.19	45.01/70.44	30.56/46.10	52.91	72.78
U-Net [23]	69.33/85.03	67.47/90.51	54.82/77.33	51.37/72.32	48.68/71.79	34.68/47.14	54.39	74.02
DeepLab V3+ [26]	70.90/88.31	68.27/90.95	58.66/78.72	55.50/74.69	51.81/75.99	44.19/53.28	58.22	76.99
NLNet [28]	73.27/94.50	69.23/95.33	58.01/84.24	54.96/81.30	58.93/82.71	38.36/57.99	58.79	82.68
OCNet [46]	73.95/95.31	69.01/96.16	61.11/84.96	55.81/81.99	57.71/83.42	36.68/58.49	59.04	83.39
ResUNet-a [42]	76.42/93.57	72.07/97.74	62.17/87.34	57.45/84.30	61.84/83.96	42.04/55.27	62.00	83.70
SCAttNet [51]	78.66/95.74	**73.41**/**98.16**	**64.74**/88.44	59.55/89.42	61.67/86.16	42.28/56.98	63.38	85.81
HMANet [53]	77.24/93.85	72.03/97.56	62.63/88.65	58.97/89.12	63.06/89.84	50.30/66.13	64.04	87.53
LANet [52]	78.11/95.06	72.9/97.47	64.28/87.81	59.13/88.79	61.24/85.55	41.98/56.57	62.49	84.61
DANet [29]	72.90/95.12	69.53/95.98	60.64/85.68	54.84/82.69	59.05/84.13	42.01/58.98	59.83	83.76
SLMFNet (ours)	**79.06**/**96.68**	73.06/98.13	64.23/**90.55**	**61.87**/**91.12**	**64.69**/**89.97**	**50.24**/**68.84**	**65.53**	**89.22**

DeepLab V3+ demonstrates a notable improvement, achieving an increase of approximately 3% in OA compared to U-Net by enlarging the local receptive field. This initial enhancement suggests the benefits of leveraging contextual information. Subsequently, attention-based models further advance this concept by enabling the network to capture long-range and short-range contextual information comprehensively. NLNet, as the pioneering self-attention model in computer vision, showcases a remarkable advancement, outperforming DeepLab V3+ by nearly 6% in OA. This substantial improvement highlights the effectiveness of self-attention mechanisms.

Following this trend, OCNet takes a step further by incorporating contextual object dependencies in addition to pixel-wise correlations, resulting in a modest accuracy boost. Similarly, DANet achieves accuracy levels on par with NLNet and OCNet, signifying its competitiveness. However, it is essential to note that specialized RSI-specific networks exhibit significant potential when considering the intricate and diverse properties of RSIs. This inherent complexity contributes to the outstanding accuracy achieved by models such as ResUNet-a, SCAttNet, HMANet, and LANet.

Moreover, variants of attention mechanisms, including SCAttNet, HMANet, and LANet, demonstrate superiority over the hybrid ResUNet-a. Specifically, HMANet stands out, delivering a performance increase of over 4% through the integration of multiple attention mechanisms. SLMFNet also leverages attention mechanisms, enabling the attentive learning of spatial and channel dependencies. This trait not only ensures performance at least on par with SCAttNet and HMANet but also enriches contextual cues. Furthermore, with the incorporation of MLFD, SLMFNet surpasses the 90% and 91% accuracy thresholds for the recognition of easy-to-distinguish and frequently occurring pixels.

Among all categories, SLMFNet achieves the highest accuracy in five out of six scenarios. For the segmentation of challenging objects like low vegetation and trees, SCAttNet and HMANet achieve accuracies of 89% and over 89%, respectively. In contrast, thanks to the fusion of multi-level features, SLMFNet consistently surpasses 90% and 91% accuracy levels for these categories. Similar trends are observed for objects like cars, which are often characterized by indistinct boundaries.

**Fig 9:**
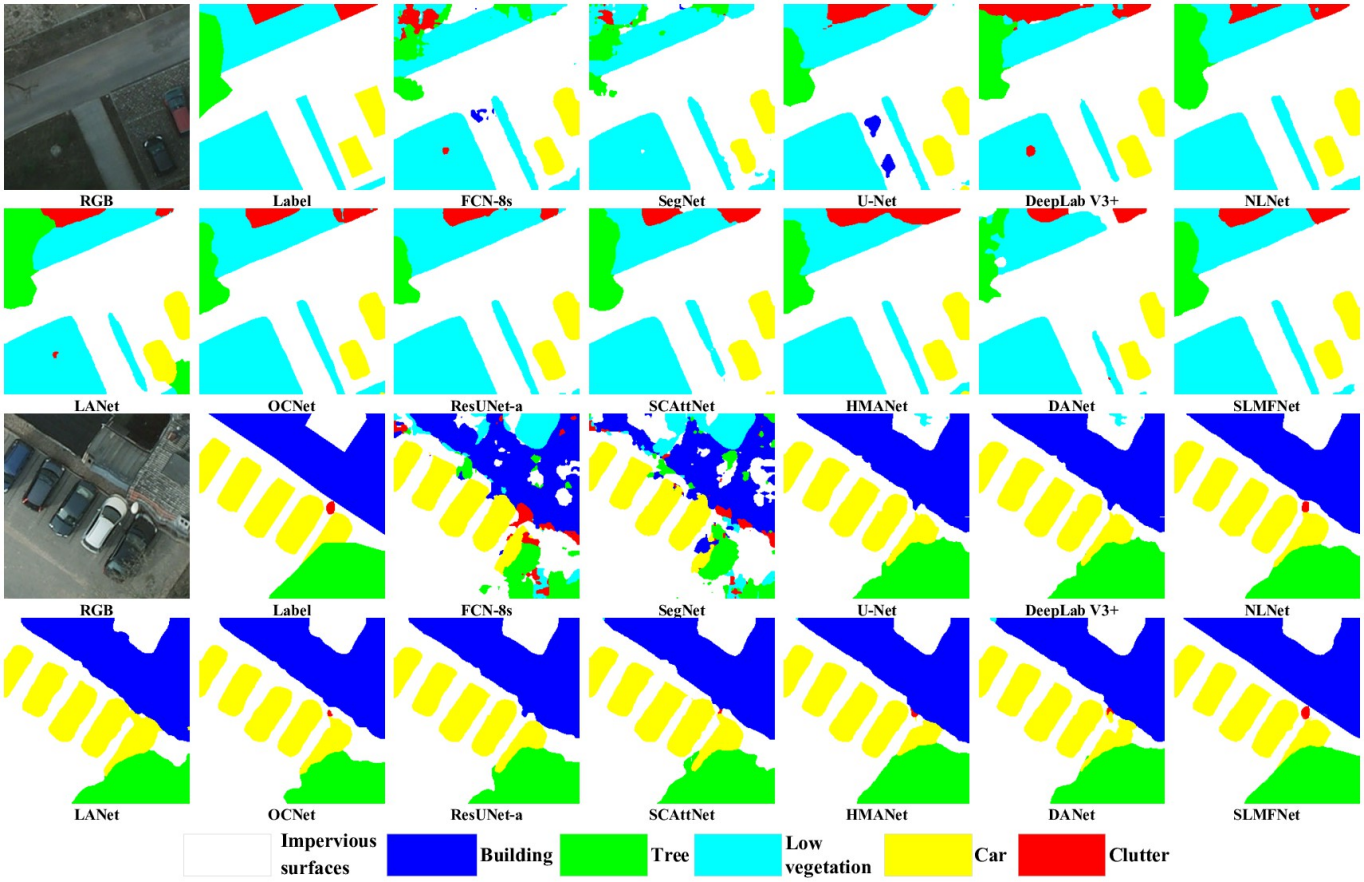
Visual inspections of random samples from the Potsdam test set.

In addition to numerical indicators, visualization is another way to evaluate the performance appreciably. Fig 9 presents two random samples from the Potsdam test se. Closer inspection of the Figure shows that SLMFNet segments the raw image in the highest consistency with the ground truth mask. Whatever the pixel belongs to stuff or objects, the multi-attentive representations and the multi-level feature fusion make the pixel classified indeed. As a result, the dense predictions are overall superior. As clearly presented, conventional models, such as FCN-8s. SegNet, and U-Net, are susceptible to be interfered with by the intra-class variants and inter-class similarities. For example, some road parts are labeled as buildings, and the edges between low-vegetation and tress are blurry. SLMFNet consolidates the discriminative and distinguishable representations by losslessly fusing multi-level features. Eventually, the uniformity and coherence of our results are the best.

In summary, SLMFNet takes an entire account of the imaging properties and visual characteristics of aerial imagery. Experimental results provide compelling evidence that selectively learning contextual affinity then losslessly fusing multi-level features can significantly boost segmentation performance.

#### 4.4.2 Results of DeepGlobe dataset

Unlike aerial images, satellite imagery has a lower spatial resolution, covers a broader observation range, contains more diverse stuff and entities, and has more complex imaging conditions. Hence, it is more challenging to produce pixel-level semantic masks of satellite imagery. Aiming at assessing the generalizability and stability, another experiments are conducted on the DeepGlobe dataset.

The results are reported in Table 5 manifests an analogous tendency. Though the data properties are changed, the visual essence of stuff and objects are consistent. Predominantly, SLMFNet attains the highest scores except for class-wise accuracy of forest land. This minor fluctuation is legitimate due to the random initialization of the network. Generally speaking, attention-based networks perform better than fundamental ones. And RSI-specific models provide finer labels, especially the models that design and integrate AMs.

**Table 5:** The results of the DeepGlobe test set with class-wise performance in form of IoU/OA and overall performance with mIoU and OA, where bold indicates the best.

Methods	Urban land	Agriculture land	Rangeland	Forest land	Water	Barren land	Unknown	mIoU	OA
FCN-8s [21]	41.82/64.89	48.32/74.98	41.12/63.82	40.35/62.61	49.71/77.15	36.39/56.47	34.56/53.63	41.75	64.79
SegNet [22]	45.27/70.25	52.31/81.17	44.52/69.09	43.68/67.78	53.82/83.52	39.39/61.13	37.41/58.06	45.20	70.14
U-Net [23]	49.32/76.54	55.20/85.66	48.51/75.28	47.59/73.86	54.87/85.15	38.16/59.22	37.42/58.07	47.30	73.40
DeepLab V3+ [26]	49.77/77.23	55.53/86.18	50.02/77.62	47.99/74.48	56.15/87.14	42.07/65.29	39.38/61.11	48.70	75.58
NLNet [28]	51.50/79.92	56.40/87.52	51.53/79.97	49.37/76.61	56.75/88.06	43.14/66.95	40.98/63.59	49.95	77.52
OCNet [46]	52.02/80.73	56.93/88.35	52.26/81.10	50.20/77.91	57.56/89.33	43.87/68.08	42.25/65.57	50.73	78.72
ResUNet-a [42]	50.92/79.03	58.08/90.13	51.34/79.67	51.50/79.92	56.84/88.21	49.63/77.02	45.68/70.88	52.00	80.69
SCAttNet [51]	50.51/78.69	57.60/89.74	50.91/79.32	53.08/82.70	60.21/93.79	46.38/72.26	45.22/70.45	51.99	80.99
HMANet [53]	50.71/79.01	57.83/90.09	51.12/79.64	53.29/**83.02**	60.44/94.16	46.56/72.55	45.4/70.73	52.19	81.31
LANet [52]	50.15/78.44	57.19/89.46	50.56/79.08	52.7/82.44	59.77/93.5	46.05/72.04	44.9/70.23	51.26	80.17
DANet [29]	51.22/80.11	56.05/87.67	50.92/79.65	48.84/76.39	56.79/88.83	43.18/67.54	40.71/63.67	49.67	77.69
SLMFNet (ours)	**57.51**/**82.56**	**65.59**/**92.16**	**57.98**/**83.23**	**58.16**/81.49	**64.34**/**94.36**	**56.05**/**80.47**	**51.58**/**72.05**	**58.75**	**83.76**

As reported by the measurable indicators, all the RSI-specific networks preferably actualize dense predictions with more than 50% and 80% of mIoU and OA, while fundamental networks drop below 50% and 76%. The land cover types covered by satellite sensors are comparatively coarse in spatial details, which is a flagrant contrast to aerial images. Therefore, the utilization of informative context is essential for enhancing the geo-objects’ details, making the pixels easily identifiable.

**Fig 10:**
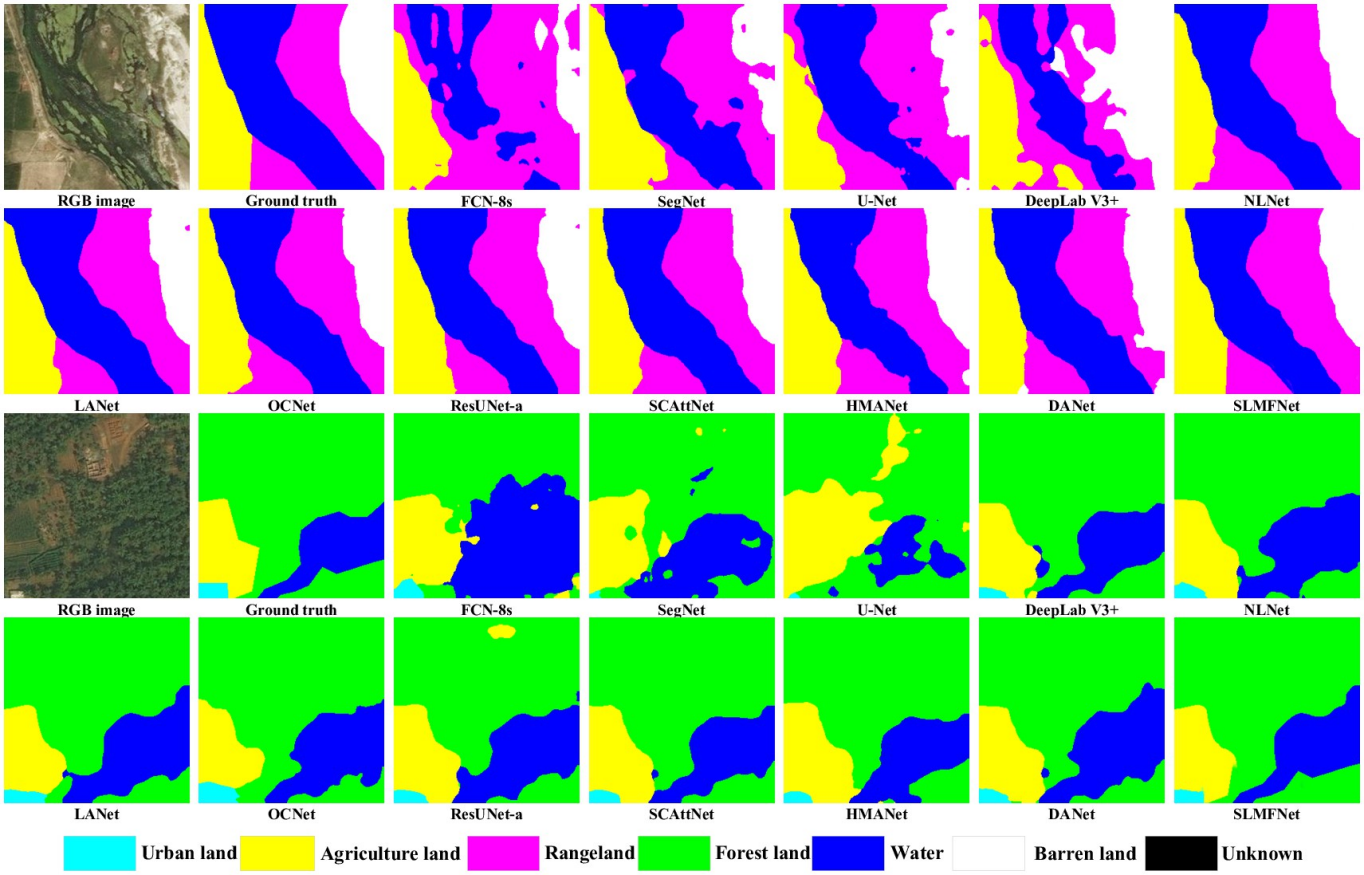
Visual inspections of random samples from the DeepGlobe test set.

Aside from numerical indicators, the visualizations in Fig 10 explicitly compare the models. Forestland comprises densely vegetated areas with diverse tree species. It encompasses regions with abundant vegetation, including forests, woodlands, and jungles. Notable features encompass variations in vegetation color, texture, and density. Barrenland denotes regions with minimal or absent vegetation, typically in arid, desert, or rocky terrains. Prominent features encompass the absence of visible vegetation, prevalence of sandy or rocky textures, and a uniform appearance. Bodies of water, including lakes, rivers, ponds, and oceans, are characterized by their distinct blue coloration and the presence of ripples or reflections, depending on surface conditions. Due to the category diversity, network-learned features should adapt to various ground objects. Simultaneously acquiring spatial and channel contextual affinities will enhance the distinguishability of representations. The raw images tell us that the objects and stuff are visually ambiguous. Therefore, the difficulty is relatively hard in segmenting. For water areas, striking differences are made by different models. RSI-specific methods act well in delineation, among which the incorporation of AMs is profitable. Together with the multi-level feature fusion, SLMFNet can segment stuff and objects with high certainty.

In conclusion, the numerical metrics and visual inspections authenticate that SLMFNet succeeds in segmenting satellite imagery. SLMFNet enables the network to surmount the salient intra-class variants and inter-class similarities with the informative context and multi-level representations.

### 4.5 Effects of selective attention modules

SAMs are devised to boost the efficiency in capturing spatial and channel dependencies. To make a fair comparison, the MLFD is removed. Instead, we adopt the standard symmetrical decoder, bringing into correspondence with DANet. And we note this version as SLMFNet v1. Intuitively, SLMFNet v1 employs the same decoder with DANet. The unique difference is the AM. The following sub-sections will discuss the variation trends of efficiency and accuracy.

#### 4.5.1 Accuracy

We first collect the mIoU and OA on the test set. As shown in Table 6, SLMFNet v1 performs better on the DeepGlobe dataset while slightly worse on Potsdam than DANet. To our best of knowledge, the Potsdam dataset consists of aerial imagery, which is visually more compact than DeepGlobe. Therefore, resampling spatial anchors cause certain losses, making the produced contextual affinity incomplete. Consequently, SLMFNet v1 drops the OA and mIoU with about 0.13% and 0.9%, practically ignorable. In contrast, the DeepGlobe dataset is particularly sparse. Therefore, the resampled anchors are sufficient for capturing and injecting arbitrary-range correlations without inducing irrelevant information. In response, SLMFNet v1 increases the mIoU and OA with about 3.3% and 2.6%.

**Table 6:** Accuracy comparisons in form of mIoU/OA on test set.

Models	SLMFNet v1	DANet
Potsdam	59.29/83.65	59.83/83.76
DeepGlobe	51.33/79.71	49.67/77.69

**Fig 11:**
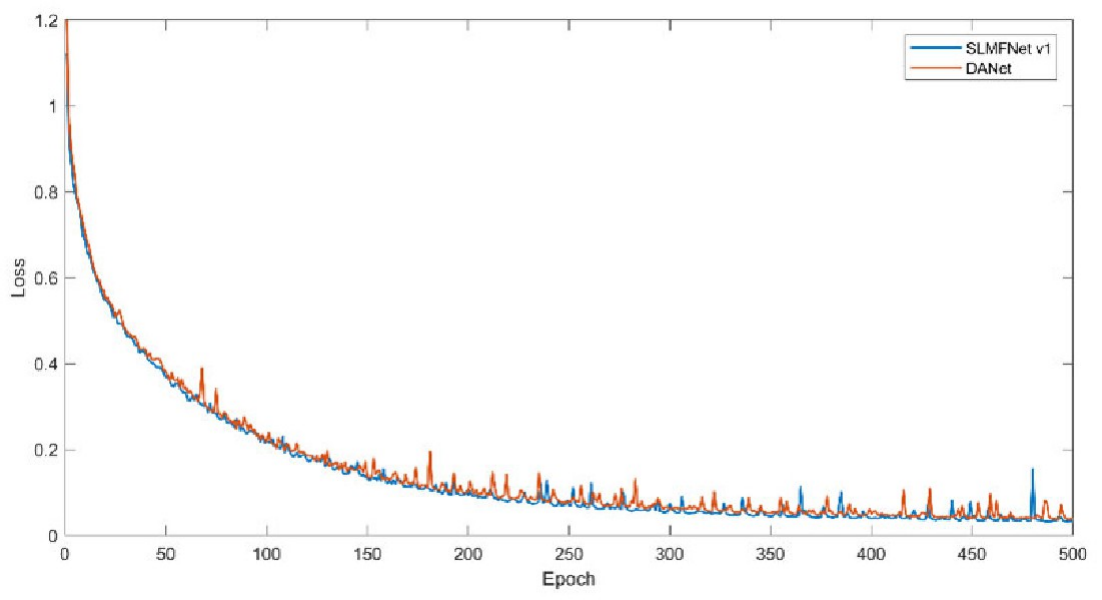
Training loss on Potsdam dataset.

**Fig 12:**
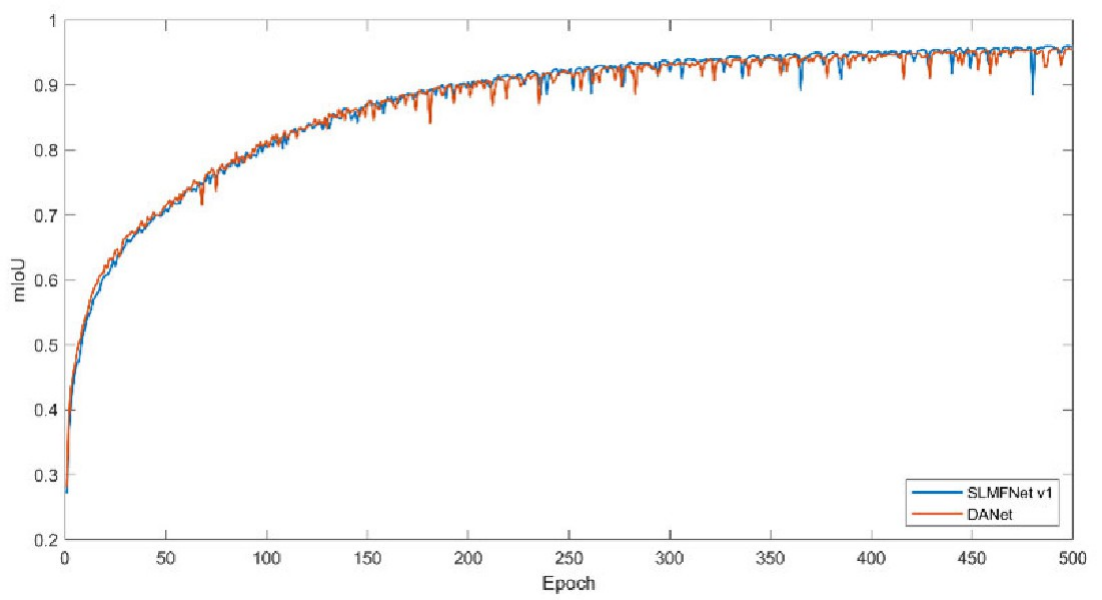
Training mIoU on Potsdam dataset.

**Fig 13:**
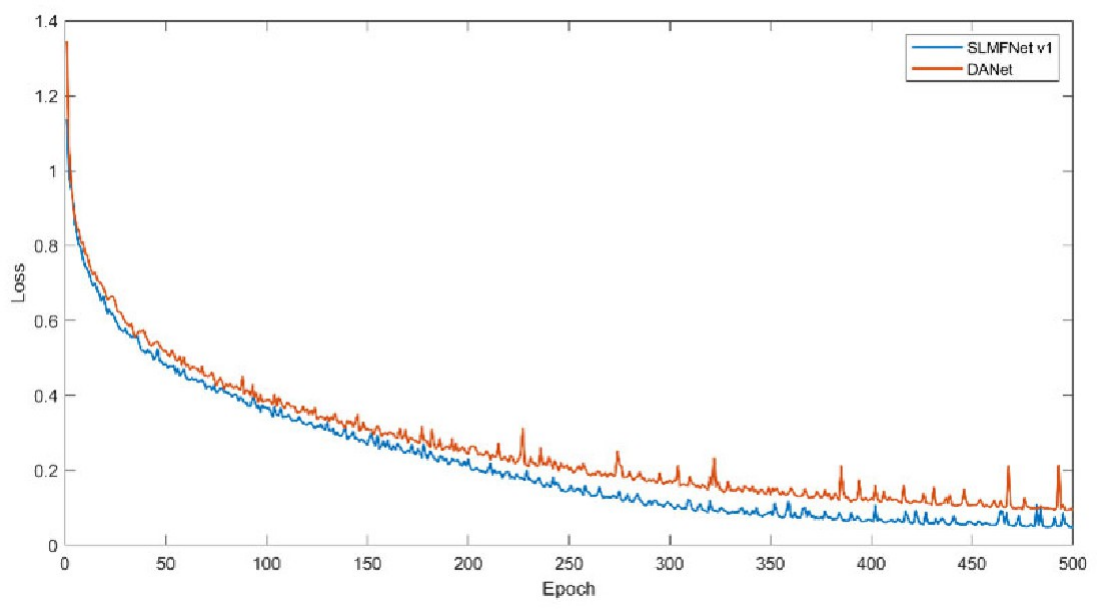
Training loss on DeepGlobe dataset.

**Fig 14:**
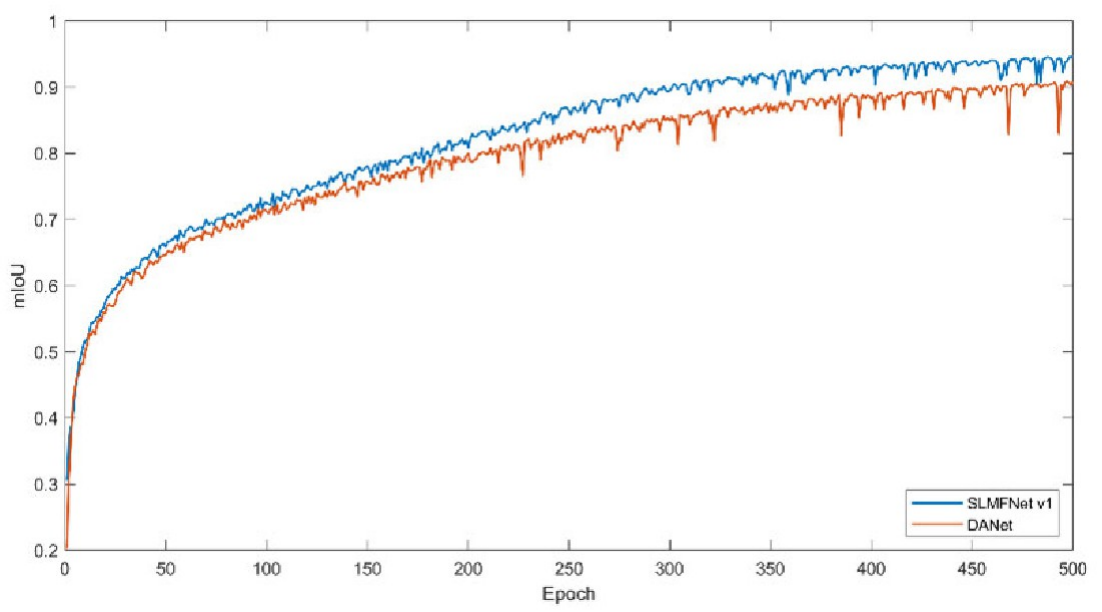
Training mIoU on DeepGlobe dataset.

Secondly, we monitor the training loss and training mIoU. Figs 11 and 12 plot the change of training loss and mIoU. Visibly, SLMFNet v1 is in line with DANet. As for training loss, at the 500 epoch, DANet drops the loss to 0.0332 whilst SLMFNet v1 decreases to 0.0386. The mIoU of the training set also provides the same characteristics. SLMFNet v1 eventually reaches 95.55%, while DANet is slightly higher with 95.84%. Unquestionably, the results are not significantly degraded. Figs 13 and 14 draw the tendency of DeepGlobe. Due to the highly sparse distribution, the selective AMs avoid importing irrelevant contextual information. As can be seen, lower loss and higher mIoU are brought by SLMFNet v1 than DANet. The mIoU experiences a rise from 90.80% to 94.66%.

Thus, we conclude that selectively learning contextual affinity hardly causes performance degradation. Specifically, for the RSI with high sparsity, SAMs work better by capturing and injecting dependable and closely associated affinity.

#### 4.5.2 Efficiency

In this part, the time cost is compared to evaluate the efficiency. In this research, we utilized a sole Nvidia A40 GPU boasting 40GB of memory for the implementation of all models. The training time per epoch and test time per sub-patch with a spatial size of 256 × 256 are collected in Table 7. The training time is an average of 500 epochs and the test time of a single image is an average of the whole test set.

**Table 7:** Efficiency comparisons.

Datasets	Phases	SLMFNet v1	DANet
Potsdam	Taining per epoch	203±16 s	262±21 s
	Test single sub-patch	23.6 ms	28.1 ms
DeepGlobe	Taining per epoch	1010±32 s	1327±41 s
	Test single sub-patch	25.5 ms	31.3 ms

By reducing the matrix manipulations, SAMs help the network execute less training and test time. For Potsdam dataset, SLMFNet v1 averagely costs 203s per epoch, while DANet needs about 262s. When inferring a single image with the same spatial size, SLMFNet v1 reduces about 4.4 ms.

The time cost on DeepGlobe is comparatively higher because of the large volume of data. However, compared to DANet, the time cost per epoch cuts down by more than 200 seconds on average. Concerning inference of single image, SLMFNet v1 boosts with more than 5 ms.

Furthermore, we perform a comparison of parameter siize and FLOPs. As depicted in Table 8, the SAM design has resulted in a reduction of approximately 24% in parameters. In terms of FLOPs, SLMFNet v1 also demonstrates competitive performance when compared to DANet. The reduction of redundant computations leads to a significant improvement concerning parameter size and FLOPs.

**Table 8:** Parameter comparisons.

Methods	Parameter (M)	FLOPs(G)
DANet	81.26	75.04
SLMFNet v1	61.65	56.93

Overall, the experimental results thoroughly corroborate the superiority of SAMs in boosting efficiency.

### 4.6 Effects of multi-level feature fusion with data-dependent upsampling

In this subsection, two counterparts are designed to validate the effects of MLFD. The first one is one-step bilinear upsampling with a ratio of 16 (corresponding to *F*_*d*_(*i*)), termed as SLMFNet with OneUP. Secondly, we replace DUpsampling by bilinear upsampling while keeping the pipeline of Fig 6, termed as SLMFNet with MultiUP.

As reported in Table 9, the comparative analysis of the SLMFNet model’s performance with its OneUP and MultiUP variants on the Potsdam and DeepGlobe datasets reveals critical insights into the efficacy of semantic segmentation techniques. The standard SLMFNet consistently outperforms its variants, indicating an inherent robustness in its architectural design. This superiority is particularly pronounced in the mIoU scores, a metric that signifies the model’s precision in classifying individual pixels into correct semantic categories. For instance, on the Potsdam dataset, the standard SLMFNet achieves a mIoU of 65.53%, compared to 61.38% and 63.60% for its OneUP and MultiUP counterparts, respectively. This trend is similarly observed in the DeepGlobe dataset, where the standard SLMFNet attains a mIoU of 58.75%, significantly higher than the other two variants. The OA metric follows a similar pattern, reinforcing the model’s general effectiveness across diverse geographical and environmental contexts.

**Table 9:** Results of different decoders in form of mIoU/OA.

Models	SLMFNet with OneUP	SLMFNet with MultiUP	SLMFNet
Potsdam	61.38/85.93	63.60/85.86	65.53/89.22
DeepGlobe	50.95/79.70	53.34/82.78	58.75/83.76

The results from the Potsdam and DeepGlobe datasets highlight the standard SLMFNet’s adaptability and efficiency in dealing with varying complexities inherent in different types of aerial and satellite imagery. The noticeable improvement in both mIoU and OA by the standard SLMFNet suggests that its internal mechanisms are better suited for capturing and differentiating between the nuanced features of diverse land cover categories. This is particularly crucial in remote sensing applications where accuracy in pixel classification directly impacts the practical utility of the segmentation results. The less pronounced performance of the OneUP and MultiUP variants could be attributed to possible limitations in their upscaling or feature integration processes, which may not capture the full spectrum of spatial and contextual information as effectively as the standard SLMFNet. These findings underscore the importance of a model’s internal architecture in determining its segmentation proficiency, especially in complex and varied environments such as those represented by the Potsdam and DeepGlobe datasets. Further research could delve into dissecting the specific architectural elements of the SLMFNet that contribute to its enhanced performance, offering valuable insights for future advancements in the field of semantic segmentation.

## 5 Conclusions

Semantic segmentation is vital for intelligent interpretation of RSIs in land cover classification. Acquiring contextual information is essential for obtaining distinctive representations. Previous research demonstrates the effectiveness of AMs in capturing contextual dependencies across domains. However, these attention-based models often treat both pixels and channels equally, leading to the introduction of irrelevant and intrusive associations. This is primarily due to the sparse distribution, high intra-class diversity, and inter-class similarity in RSIs, which substantially differ from natural images, ultimately increasing computational complexity.

To address these challenges, we introduce SLMFNet, a novel segmentation network that efficiently refines learned representations and seamlessly fuses multilevel features. We first employ SAMs to learn contextual affinities across spatial and channel domains with fewer matrix operations. Subsequently, a multilevel feature fusion decoder with learnable DUpsampling is designed to gradually merge and recover densely predicted feature maps. SLMFNet is subjected to extensive comparative analysis on two benchmarks, showcasing its competitive and compelling performance in both quantitative and qualitative assessments.

In conclusion, the advancements presented in this study have significant implications for practical applications in a variety of real-world scenarios. The enhanced accuracy and efficiency in semantic segmentation of remote sensing images offered by our approach can be instrumental in fields such as urban planning, environmental monitoring, disaster response, and agricultural management. For instance, in urban development, our method can aid in precise mapping and analysis of land use, supporting sustainable urban planning decisions. In the context of environmental monitoring, it can contribute to more accurate assessments of land cover changes, aiding in the conservation of natural resources. Additionally, in disaster management, the ability to rapidly and accurately segment images can be crucial in assessing damage and guiding rescue operations. In agricultural settings, our approach can assist in monitoring crop health and land conditions, promoting efficient and sustainable agricultural practices. These applications underscore the potential of our research to not only advance the academic understanding of remote sensing image processing but also to contribute tangible benefits to society by informing policy and decision-making processes. By bridging the gap between theoretical research and practical implementation, we aim to pave the way for future innovations that harness the full potential of semantic segmentation in addressing real-world challenges.

Nonetheless, three aspects necessitate further exploration and enhancement. Firstly, the manually set SPP ratios and compressed channel numbers can be adjusted by the network based on learned data, prompting additional research. Secondly, as SLMFNet demands ample well-annotated data for training, the creation of a cost-effective semi-supervised variant holds promise. Moreover, the availability of multi-modal RSIs could be considered to achieve accurate LCC, especially exploring models for few-shot learning paradigms. Looking ahead, we anticipate the development of an effective and accurate land cover classification approach with data adaptability.
